# Weakening of interaction networks with aging in tip-link protein induces hearing loss

**DOI:** 10.1042/BCJ20200799

**Published:** 2021-01-13

**Authors:** Surbhi Garg, Amin Sagar, Gayathri S. Singaraju, Rahul Dani, Naimat K. Bari, Athi N. Naganathan, Sabyasachi Rakshit

**Affiliations:** 1 Department of Chemical Sciences, Indian Institute of Science Education and Research Mohali, Punjab, India; 2 Centre de Biochimie Structurale INSERM, CNRS, Université de Montpellier, Montpellier, France; 3 Department of Biotechnology, Bhupat and Jyoti Mehta School of Biosciences, Indian Institute of Technology Madras, Chennai 600036, India; 4 Institute of Nano Science and Technology (INST), Phase-10, Sector-64, Mohali, Punjab 160062, India; 5 Centre for Protein Science Design and Engineering, Indian Institute of Science Education and Research Mohali, Punjab, India

**Keywords:** ARHL, circular cross-correlation, interaction networks, protein conformation, statistical and computational modeling, tip-link proteins

## Abstract

Age-related hearing loss (ARHL) is a common condition in humans marking the gradual decrease in hearing with age. Perturbations in the tip-link protein cadherin-23 that absorbs the mechanical tension from sound and maintains the integrity of hearing is associated with ARHL. Here, in search of molecular origins for ARHL, we dissect the conformational behavior of cadherin-23 along with the mutant S47P that progresses the hearing loss drastically. Using an array of experimental and computational approaches, we highlight a lower thermodynamic stability, significant weakening in the hydrogen-bond network and inter-residue correlations among β-strands, due to the S47P mutation. The loss in correlated motions translates to not only a remarkable two orders of magnitude slower folding in the mutant but also to a proportionately complex unfolding mechanism. We thus propose that loss in correlated motions within cadherin-23 with aging may trigger ARHL, a molecular feature that likely holds true for other disease-mutations in β-strand-rich proteins.

## Introduction

The origin of age-related hearing loss (ARHL) or presbycusis is multifactorial [1–4]. However, the damage to the inner ear due to environmental and physiological noise is considered to be one of the significant causes of ARHL [1,5]. In the inner ear, the sound stimuli is amplified as well as mechano-transduced into an electrical signal by controlled deflections of stereocilia that are atop of hair-cells [6,7]. Tip-links that are formed by two long-chain non-classical cadherins, cadherin-23 (Cdh23) and protocadherin-15 (Pcdh15), connect the tips of two adjacent stereocilia and act as force-conveying gating-springs facilitating the mechanotransduction [8,9]. Cdh23 in tip-links is identified as one of the ARHL (*ahl1*) loci in inbred mice models [10]. Thus, environmental noise-induced perturbations in the structure–function of Cdh23, with aging or biochemical modifications, affect the mechanotransduction adversely and contribute to the hearing loss diseases, including ARHL [10–14]. Screening of random mutations on Cdh23 in mice pointed to a specific single-point mutation of serine 47 (Ser47) to proline (S47P) in the outermost extracellular (EC1) domain contributing to progressive hearing loss (PHL) [15]. PHL is an aggressive form of ARHL where the onset of hearing loss initiates within 3 months rather than the usual age of 9–12 months for ARHL in mice [15]. For humans, the hearing loss age varies between 8 and 16 years in the case of PHL [16,17]. In this regard, it is interesting to note that the entire sensory machinery for hearing is perfectly developed in PHL, and so the mechanoresponsive features in response to sound stimuli. A loss in hearing ability in PHL emerges only with maturation, though sooner than the usual ARHL. We, therefore, hypothesized that PHL is an early version of ARHL and can serve as a biophysical model for ARHL.

Mis-sense mutations that cause diseases or those that play a significant role in the development of disease phenotype majorly involve charge neutralization, charge reversal or reversal of polarity [18]. For transmembrane proteins, the frequency of non-polar to non-polar conversions is equally frequent among disease-associated mutations. Among all mutations to proline, serine to proline is the second most-frequent in disease associative mutations in transmembrane proteins [19]. For other proteins, mutation of serine is less frequent. While the mutation of proline to serine is still reported, mutation from serine to proline is not frequent, and its association with diseases is rare [20,21]. How do mutations to proline lead to hearing loss progression is thus an important aspect to decipher. In general, proline mutations result in complex and context-dependent kinetic, structural, and functional outcomes [22]. While the stereochemical rigidity in the pyrrolidine ring increases protein stability by reducing the conformational entropy of the unfolded state, the imide group of proline often destabilizes proteins due to the loss of main-chain hydrogen bonds and hydrophobic interactions [23]. The resulting delicate balance between the entropic free energy changes from the stereochemical rigidity of pyrrolidine and the enthalpic energy loss from the fewer intramolecular interactions determines the thermodynamic stability effect of proline substitution in proteins. The effect of proline on the folding kinetics of proteins is equally complex. Peptidyl-prolyl cis-trans isomerization in *in vitro* folding experiments often results in distinct phases that can, in turn, slow down folding [24]. On the other hand, proline could also equally affect the native state dynamics thus contributing to non-intuitive emergent effects driven by the weak non-covalent and pliable nature of the intramolecular interaction network [25].

**Figure 1.**
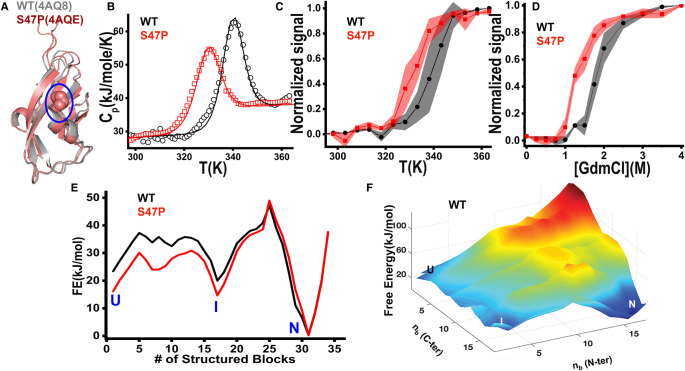
Equilibrium studies and statistical modeling. (**A**) Ribbon diagrams of Cdh23-EC1 WT (4AQ8) and S47P (4AQE). P47 is marked in spheres and encircled in blue. (**B**) Scanning calorimetry profiles (circles) together with fits from the bWSME model (solid lines). (**C**,**D**) Thermal (**C**) and chemical unfolding (**D**) of cadherin variants as monitored by far-UV CD and intrinsic tryptophan fluorescence, respectively. The data are normalized to estimate unfolded populations (see Methods). The shaded portions (black (WT) and red (S47P)) mark the standard error of mean (SEM) from experimental repeats. (**E**) One-dimensional free energy profiles predicted by the bWSME model highlighting the unfolded (U), intermediate (I), and native states (N). (**F**) Free energy landscape of the WT from the bWSME model as a function of the number of structured blocks in the N- or C-terminus.

Mutations in the extracellular regions do not affect the cellular signaling. Moreover, the stereochemical alterations in Cdh23 caused by S47P mutation do not contribute to any obvious structural change that can destabilize the tip-link complex [15,26]. Specifically, the corresponding X-ray crystal structures of the wildtype (WT) (4AQ8) and mutant (S47P) (4AQE) reveal a C_α_ and all-atom RMSD of 0.62 Å and 0.65 Å, respectively [26]. *In vitro* measurements of the binding-affinity of the tip-link complex [26] or the mechanoresponsive behavior of the complex do not result in any significant change on S47P mutation (Supplementary Figure S1). These observations raise questions on the molecular mechanisms through which the S47P mutation contributes to hearing loss at an early age. Since there is no difference in the stability of the tip-link complex which involves two outermost extracellular (EC) domains, here we focus our study on the most extracellular domain (i.e. Cdh23-EC1, Figure 1A) where the mutation is present. We address this question through an array of quantitative ensemble measurements, theoretical modeling, all-atom molecular dynamics (MD) and steered MD simulations that interrogate the mechanical stability, thermodynamic stability, dynamics and kinetics of the mutant compared with the WT. Contrary to the disease mutation, S47 is evolutionarily replaced by Val (V47) (Supplementary Figure S2) in some vertebrates imparting a significantly better sensitivity in hearing at low-frequency sound than humans [27,28]. To highlight the contextual effect of the disease mutation, we compare the properties of V47-variant with the other two variants. Overall, we show how multiple properties of the protein are affected through a single-point mutation thus providing a glimpse of the molecular origins of disease phenotype, with application to progressive hearing loss.

## Materials and methods

### Protein expression and purification

Three variants of the outermost extracellular domain EC1 (Q24 to D124) of mouse Cdh23 (NP_075859.2) — WT, S47P and V47 — are cloned in pET21a vector (Novagen) between Nde1 and Xho1 restriction sites. We considered the Q24 as Q1 in our experiments as residues 1–23 are signal peptides and are cleaved off after anchoring to the membrane. The recombinant proteins are expressed in *E. coli* BL21 Codon Plus (DE3)-RIPL (Stratagene, U.S.A.) cells as reported previously [26]. The proteins are first affinity-purified using Ni-NTA beads (Qiagen) and refolded from 8 to 0 M urea buffer using a reported refolding protocol [29]. Finally, the refolded fractions are re-filtered using size exclusion chromatography in 25 mM HEPES 25 mM KCl, 100 mM NaCl, and 2 mM CaCl_2_ (pH 7.5) buffer using Superdex 200 column (GE Healthcare) (Supplementary Figure S3c).

### Differential scanning calorimetry

Differential scanning calorimetry (DSC) experiments are carried out in 1 mM HEPES buffer at pH 7.4 containing 10 mM NaCl and 2 mM CaCl_2_ in a Microcal VP-DSC instrument (Malvern, U.K.). Several water–water and buffer–buffer scans are acquired before the protein scan to equilibrate the cells. The protein scans are acquired at a concentration of ∼11.2 μM and ∼15.1 μM for the WT and S47P mutant, respectively, at a scan rate of 1 K/min. The absolute heat capacity is calculated using the method of Sanchez-Ruiz and co-workers [30].

### Circular dichroism (CD)

CD spectra for the proteins are collected in CD buffer (1 mM HEPES, 10 mM NaCl, and 2 mM CaCl_2,_ pH 7.5) using Chirascan (Applied Photophysics) spectrophotometer attached with a Peltier system. The FUV CD spectra for all variants show two troughs as expected, negative peak maxima at ∼230 nm, typical feature for β-turns and a relatively weaker trough at ∼215 nm for β-strands (Supplementary Figure S3a). The temperature unfolding experiments are carried out using 25 μM of proteins and the temperature is varied from 295 to 365 K at an interval of 5 K. The spectra are collected from 260 to 205 nm in a quartz cuvette with a path length of 1.0 mm. The mean residue ellipticity (MRE) at a wavelength of 215 nm is plotted against temperature and the fraction of unfolded protein with temperature is calculated using the following equation:

1 $$\alpha =\frac{\left(\theta_{T}-\theta_{u}\right)}{\left(\theta_{u}-\theta_{f}\right)}$$

where *α* is the fraction of unfolded protein, *θ_T_* is the observed ellipticity at any temperature, *θ_f_* is the ellipticity of the native protein and *θ_u_* is the ellipticity of the unfolded protein. *T_m_* is estimated from the peak-maximum of the normalized first derivative plot of *α* w.r.t. temperature.

### Steady state intrinsic tryptophan fluorescence

The thermal and chemical unfolding using fluorescence are performed in Jobin-Yvon Fluoromax-4 spectrofluorometer. In total, 10 µM of proteins are used. The intrinsic fluorescence spectra for all variants show peak maxima at 338–339 nm, appearing from a single tryptophan at the 66th position (W66). Identical fluorescence emission for all variants indicate the exposure of W66 to solvent of the same extent. (Supplementary Figure S3b). For the temperature-dependent unfolding studies, the temperature is ramped from 295 to 365K at 5 K interval and with an equilibration time of 2 min. The protein samples are excited at 295 nm and emission spectra are collected from 315 to 450 nm. Following equation (1), the fraction of unfolded protein (*F_u_*) is estimated from the red-shift in the wavelength of peak maxima and plotted with temperature. *T_m_* is obtained from the normalized first derivative of *F_u_* with respect to temperature.

For chemical denaturation, the concentration of GdmCl is varied from 0.5 to 4.0 M at 0.5 M interval and the fluorescence spectra are recorded at each concentration. Following equation (1), the fraction of unfolded protein (*F_u_*) is estimated from the red-shift in the wavelength of peak maxima and plotted with the concentration. *C_m_* is obtained from the normalized first derivative of *F_u_* with respect to [GdmCl].

### Stopped-flow kinetics measurement

Stopped-flow kinetics experiments are carried out using a Chirascan (Applied Photophysics) spectrometer, connected to a stopped-flow apparatus (SF. 3; Applied Photophysics). For refolding, the proteins in the denaturant buffer (25 mM HEPES, 25 mM KCl, 100 mM NaCl, 2 mM CaCl_2,_ 4 M GdmCl (pH 7.5)) are mixed to the refolding buffer (25 mM HEPES 25 mM KCl, 100 mM NaCl, 2 mM CaCl_2_ and xM GdnCl (*x* = 0–1.4) (pH 7.5)) at a ratio of 1 : 10. The dead time of mixing is ∼3 ms The final protein concentration is maintained at 10 µM. The samples are excited at 295 nm and fluorescence emissions are monitored using a 320 nm band-pass filter. The emission is recorded for 10 s and 10 traces are acquired for every GdmCl concentration. The average refolding time-traces are analyzed by fitting the data to a bi-exponential time-dependence from which rates and amplitudes are extracted.

### Coarse-grained pulling simulations

We used the self organizing polymer (SOP) [31] model as implemented in the program (SOP-GPU) [32] for coarse-grained pulling simulations. Briefly, this model describes each residue by a single interaction centre (at C_α_ position). The total potential energy for a conformation is given by

$$V_{T}=V_{\mathrm{FENE}}+V_{\mathrm{NB}}^{\mathrm{ATT}}+V_{\mathrm{NB}}^{\mathrm{REP}}$$

$$V_{\mathrm{FENE}}=-\overset{N-1}{\underset{i=1}{\sum }}\frac{k}{2}R_{0}^{2}\log\left(1-\frac{{\left(r_{i,i+1}-r_{i,i+1}^{\circ }\right)}^{2}}{R_{0}^{2}}\right)$$

$$V_{\mathrm{NB}}^{\mathrm{ATT}}\;=\;\overset{N-3}{\underset{i=1}{\sum }}\overset{N}{\underset{\;j=i+3}{\sum }}\varepsilon_{h}\left[{\left(\frac{r_{ij}^{\circ }}{r_{ij}}\right)}^{12}-2{\left(\frac{r_{ij}^{\circ }}{r_{ij}}\right)}^{6}\right]\Delta_{ij}$$

$$V_{\mathrm{NB}}^{\mathrm{REP}}=\;\overset{N-2}{\underset{i=1}{\sum }}\varepsilon_{1}{\left(\frac{\sigma_{i,i+2}}{r_{i,i+2}}\right)}^{6}+\overset{N-3}{\underset{i=1}{\sum }}\overset{N}{\underset{\;j=i+3}{\sum }}\varepsilon_{1}{\left(\frac{\sigma }{r_{ij}}\right)}^{6}(1-\Delta_{ij})$$

The first term, *V*_FENE_ is the finite extensible nonlinear elastic potential which describes the backbone connectivity. *r_i_*_,*i*+1_ is the distance between the neighboring residues *i* and *i*+1 with *r*^0^*_i_*_,*i*+1_ being the corresponding distance in the native state. *N* is the total number of residues in the protein. The second term, $V_{\mathrm{NB}}^{\mathrm{ATT}}$, describes the attractive potential between the residues which are closer than the defined criterion. In all the simulations, this criterion is set as any residues which have C_α_ atoms closer than 8 Å or any side-chain heavy atoms closer than 5.2 Å. The value of $\varepsilon_{h}$ is set to 1.5 kcal/mol for both WT and S47P. The third term, $V_{\mathrm{NB}}^{\mathrm{REP}}$, describes all the non-native interactions, which are repulsive.

The crystal structure of the mouse cadherin23 (cdh23) EC1 domain (PDB ID: 4AQ8) is used for the wild type (WT) protein. For S47P, the same segment is extracted from the crystal structure of the complex of Cdh23 EC12 and pcddh15 EC12 (PDB ID: 4AQE). Before starting the pulling simulations, 100 independent simulations of 20 µs each are done for both WT and S47P to generate 100 structures each, which are then subjected to pulling simulation.

The steered molecular simulations are performed by fixing the N-terminus and pulling the C-terminus with a constant speed of 2.5 µM/s in the direction of the vector joining N and C-termini. The spring constant of the cantilever is set to 35 pN/nm which is in the range of typical atomic force microscopy experiments. The forces of unbinding and contour lengths corresponding to peaks are calculated using MATLAB (MATLAB 2014a, The MathWorks, Natick, 2014) scripts. The contact maps are generated from the pulling trajectories using the program CONAN [33].

### WSME model

The WSME model is a native-centric Ising-like statistical mechanical model originally developed by Wako and Saitô [34], and later by Muñoz and Eaton [35]. It considers a binary representation of residue conformational status with *1* and *0* standing for the folded and unfolded status, respectively. Thus, the conformational landscape of an *N*-residue protein can be written as a vast array of binary strings with a total of 2*^N^* conformational states or microstates. We consider a reduced representation of a protein landscape where only states involving single stretches of folded residues (SSA for single sequence approximation), two stretches of folded residues (DSA for double sequence approximation) and DSA allowing for interactions across islands (if they are identified in the folded state), are considered as successfully employed by Eaton and co-workers [36]. For the 102 residue Cdh23-EC1 domain this would still correspond to a total of 8 847 804 microstates making it challenging to use the model in a quantitative manner. To do so, we employ the recent block approximation (i.e. the bWSME model [37]) that considers stretches of residues as blocks thus further coarse-graining the landscape but that is still physically realistic to capture multiple thermodynamic and kinetic features. For the 102 residue Cadherin and considering 3-residue blocks, the protein chain would be reduced to just 34 blocks while the total number of microstates would be brought down to 105 316, thus enabling rapid and automated fitting of DSC profiles and free-energy surface generation. Despite this minimalist representation, the model is detailed enough in that it includes contributions from van der Waals interactions, electrostatic interactions with a Debye–Hückel treatment, simplified solvation free energy function and residue-secondary structure-dependent entropic penalty [38]. The final parameters that reproduce the DSC profiles are listed in the Table 1 below. Follow parameterization, partial partition functions are accumulated to generate both free energy profiles and surfaces.

**Table 1.** WSME Model Parameters

	WT	S47P
PDB ID	4AQ8	4AQE
*ξ* (van der Waals interaction energy) (J mol^−1^ per native contact identified with a 6 Å cut-off)	−62.8	−63.45
Δ*S*_conf_ (J mol^−1^ K^−1^ per residue for all non-Glycine, non-Proline residues identified as strands/helices)	−19.5	−20.5
Δ*S*_conf_ (J mol^−1 ^K^−1^ per residue for all Glycines and non-strand/helical residues)	−25.6	−26.6
Δ*S*_conf_ (J mol^−1 ^K^−1^ per residue for all prolines)	0.0	0.0
Δ*C*_p,cont_ (J mol^−1 ^K^−1^ per native contact)	−0.68	−0.68
Ionic strength (mM)	20	20
pH	7.0	7.0

### All-atom molecular dynamics simulations

The starting structures for all-atom MD simulations are the same as those used for SMD. The simulations are performed using the CHARMM36 force field [39] with the program NAMD [40] version 2.13. The structures are solvated in an octahedral TIP3P [41] water box extending 10 Å from the surface of the protein. Bonds to the hydrogen atoms are restrained using SHAKE [42] algorithm and a time step of 2.0 fs is used. Periodic boundary conditions are applied and the electrostatic interactions are calculated using particle mesh Ewald (PME) [43] method. The structures are minimized for 5000 steps using the conjugate gradient minimization algorithm. The systems are then slowly heated to reach the temperature of 300 K over 300 ps. Finally, the systems are equilibrated for 5 ns each of canonical (NVT) and isothermal–isobaric (NPT) simulations. The equilibrated systems are simulated in NPT conditions for a total of 1 µs (1*400 ns, 1*200 ns, 3*100 ns) each for WT and S47P.

### Analysis of MD simulations

The hydrogen-bond network is created from the MD simulations using PyInteraph [44] and subsequently filtered to remove the transient hydrogen bonds which are present in less than 25% (*p*_crit_ (critical persistence calculated from the plot of size of largest cluster vs. *p*_min_ (minimum persistence))) of the trajectory. The filtered network is plotted over the structures using PyMOL [45] plugin XPyder [46]. The circular dihedral correlation coefficient [47,48] is calculated using R scripts adapted from the Bios2cor package [49]. The steps included extracting the backbone dihedral angles from the concatenated trajectories using Bio3d [50,51] followed by the transformation of the matrix of dihedral angles to a circular object and calculation of circular correlation coefficient using R package ‘circular’. The matrix of circular correlation coefficients is then filtered using Z-scores and only the correlations with Z-score more than 2.5 are retained.

## Results

### Proline mutant is thermodynamically less stable than the WT

From purely entropic considerations, the proline mutant S47P is expected to be more stable than the WT. However, ensemble thermal and chemical denaturation experiments point to the exact opposite. Specifically, we monitored the temperature-dependent changes in the hydrophobic exposure of the proteins using differential scanning fluorimetry (DSF) [52] (Supplementary Figure S4), differences in the unfolding cooperativity using DSC [53], alterations in the secondary structures using CD [54], and the tryptophan environment by following intrinsic tryptophan fluorescence from a single tryptophan at 66th position (W66) [55]. The S47P variant is found to be less stable than the WT with a melting temperature (*T_m_*) of 331.2 ± 0.5 K and 339.2 ± 0.3 K, respectively, via far-UV CD (Figure 1C). Scanning calorimetry experiments display similar differences in melting temperatures (Figure 1B) but with a broader heat-capacity profile for the WT. Given that the unfolded state heat capacities do not differ between the variants, we can safely assume that the differences in stability are not a result of unfolded ensembles, to the extent that can be inferred from DSC.

The thermal melts interestingly point to differences in melting temperatures from different experiments with a span of 1–14 K for both proteins (Supplementary Figure S5). For large proteins, the difference in melting temperature is a hallmark of multi-state transitions. The likely presence of intermediate states for both WT and mutant are further confirmed from the absence of the isoemissive and isodichroic points in equilibrium thermal unfolding monitored by fluorescence and CD, respectively. Similarly, guanidinium chloride (GdmCl) melts monitored by intrinsic Trp (W66) emission display a lower chemical denaturation midpoint (*C_m_*) for the mutant compared with the WT with a *C_m_* of 0.6 M (Figure 1D). No isoemissive points are again observed for WT and mutant variants (Supplementary Figure S6). These observations highlight that intermediates are intrinsic to the Cdh23 conformational landscape.

### Statistical modeling of the cadherin conformational landscape

A mutation can have complex effects on the folding conformational landscape modulating the number and nature of the intermediates, apart from effects on the folded and unfolded ensembles [25]. To probe for such effects we employed the block description of the Ising-like bWSME model to generate a minimal 105 316 microstate representation of the folding conformational landscape of WT and the mutant S47P [37,38]. The model contributions from van der Waals interactions, electrostatics, and differences in backbone conformational entropy (see Methods). The model is parameterized by reproducing the DSC thermograms (Figure 1B) following which the free energy profiles and conformational landscapes are generated by accumulating partial partition functions. One-dimensional free-energy profiles can be generated as a function of the number of structured blocks, the natural order parameter for this model. It reveals a multi-state folding mechanism for both proteins (Figure 1E) with at least one major intermediate (the label I in Figure 1E) at 17 structured blocks.

The most probable folding mechanism can be extracted by plotting the free-energy landscape as a function of the number of structured blocks in the N- and C-terminal half of the domain. It can be seen that the intermediate I involves significant structure in the C-terminal half of the protein (note the sea of blue in Figure 1F) following which the N-terminal half folds. The features on the unfolded side of the barrier are minimally affected, in agreement with DSC experiments that point to minimal unfolded state perturbation. These results are evidence that the mutation does not alter the nature of any partially structured state populated in the folding landscape or the unfolded ensemble but likely has an impact only on the folded ensemble. The model also predicts that the mutant would exhibit a lower folding rate as the folding thermodynamic barrier height is higher by ∼6 kJ mol^−1^ in the mutant compared with the WT (Supplementary Figure S7).

### Proline mutation induces weaker correlated motions in β-strands

**Figure 2.**
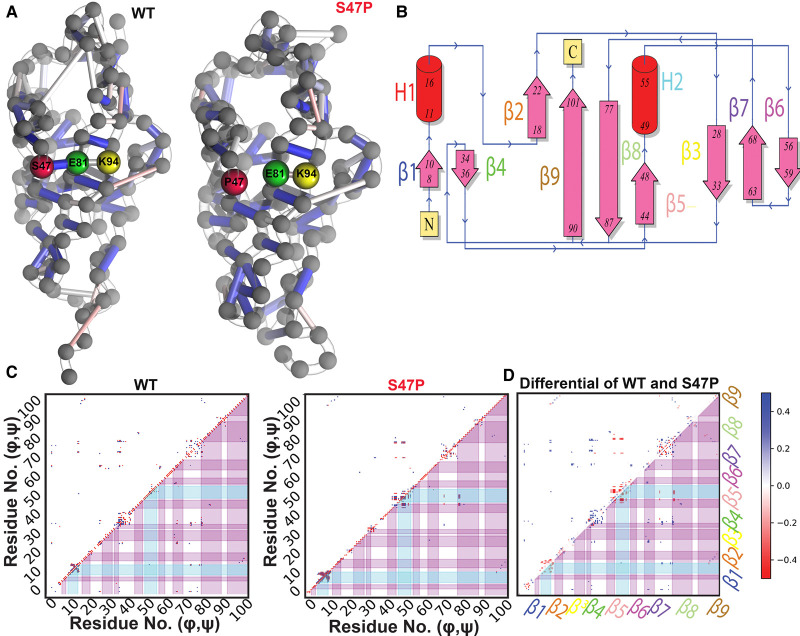
Effect of S47P mutation on the intramolecular *H*-bond network and circular cross-correlations. (**A**) Bead-and-stick representation of intramolecular *H-*bond networks in the WT (left) and S47P (right). Colored tubes indicate the *H*-bonds between residues while the colors indicate the degree of persistence (Blue (1)-White (0.62)-Red (0.25)). (**B**) Topology diagram of the Cdh23-EC1 domain. β-strands are marked as β (1–9) and helices as H (1–2). The terminals are marked as N and C. (**C**) Circular correlation coefficient maps of the main-chain dihedrals for WT (left) and S47P (right). (**d**) Differential circular correlation coefficient of the main-chain dihedrals of WT in relation to S47P. β-strands 1–9 are marked. The color map indicates the values for correlations.

The downside of the native-centric model is that it does not account for intricate mutational effects on the folded ensemble. To explore this possibility, we performed 1 µs long all-atom MD simulations in explicit solvent for both the WT and the mutant and measured the differences in the equilibrium intramolecular hydrogen-bond networks (see Methods). We find that S47 forms an integral part of the interaction network making a stable *H*-bond with E81 with 46% persistency, which in turn mediates another *H*-bond with K94 (Figure 2A). The S47P mutation eliminates this *H*-bond, and in addition to the loss of the direct interaction, weakens the *H*-bond between E81 and K94 (Figure 2A).

Long-range *H*-bond networks also control the correlated motions among β-strands in β-sheet rich proteins. Such correlated motions respond to physical perturbations by controlling the extent and rate of elastic deformations in a protein [48,56–60]. Proteins with stronger correlated motions can thus withstand larger perturbations. The correlated motions among β-strands in tip-link proteins are particularly important as they are evolutionarily designed to absorb and dissipate tension received from sound stimuli. Tip-link proteins undergo elastic conformational deformations under these high-frequency tensions from sound stimuli of varying magnitude [61–63]. Given that Cdh23-EC1 consists of nine β-strands (Figure 2B), it is possible that the rewired *H-*bond networks in S47P weaken the associated correlated motions. We thus calculated the circular cross correlation coefficient of the main-chain dihedrals (*φ* and *ψ*) between all pairs of residues for both WT and S47P proteins, as estimates of the correlated motions of the protein backbone (see Methods). We observe that in WT, the main-chain dihedrals are correlated to not just the adjoining β-strands β8 and β9, β2 and β9, β5 and β8, but also extends to the next-nearest β-strands viz*.* β8 and β2 (Figure 2C). In case of S47P, all the correlations between β-strands, except between β5 and β8, are significantly dampened, to an extent that they are not visible at the chosen level of significance (*Z*-value > = 2.5). It is interesting to note that the mutation (S47P) is not present in any of the β strands, rather in a loop connecting β5 and H2. S47 resides in β5 strand. To emphasize the loss of correlations, we have plotted the difference in the absolute values of the circular correlation coefficient for all the pairs of dihedrals (Figure 2D). It can be seen that all the correlations between the β-strands are weakened (blue). Some new correlations appear within the residues forming small helices in S47P (red), but these are localized and do not result in long-range correlated motions as seen in WT. Overall, the data indicate that a reduction in the number of interactions in the mutant adversely affects the correlated motions among β-strands (and loops) in the domain. The S47P domain thus loses the ability to dampen the deformations as fast as the WT version, likely contributing to a PHL phenotype.

### Proline mutation decelerates folding

**Figure 3.**
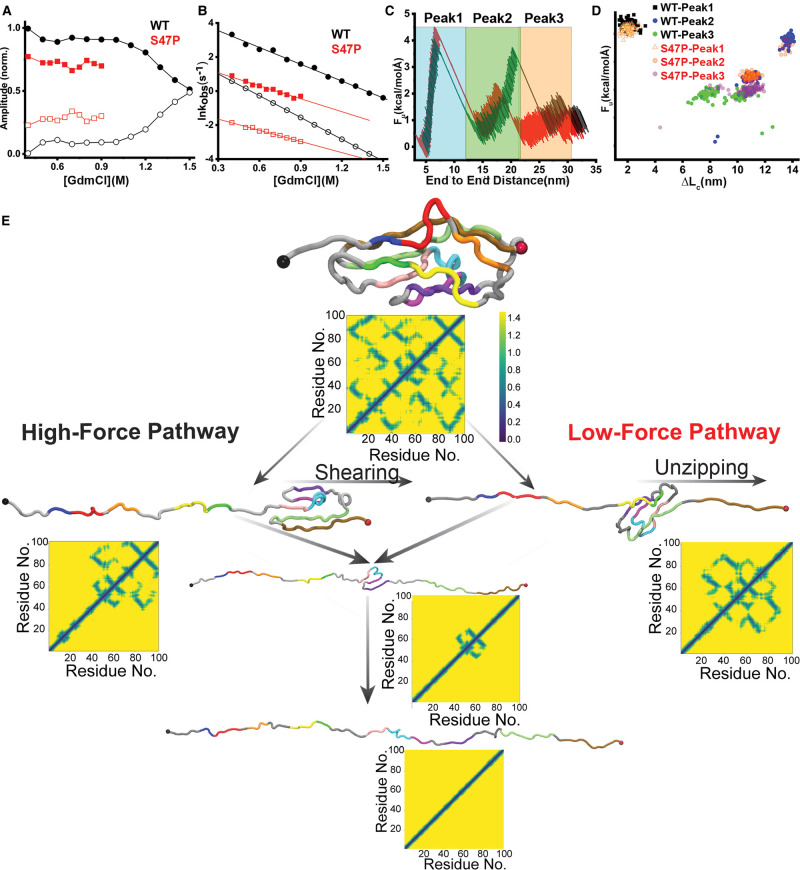
Folding and unfolding kinetic pathways for WT and S47P. (**A**) A comparison of amplitudes for the fast (closed symbols) and slow (open symbols) phases for the WT (black) and S47P (red). (**B**) Folding rates of WT (black) and S47P (red) at different GmdCl concentrations. The fast and slow components are marked as closed and open symbols, respectively. The solid lines are the corresponding linear fits to the observed rates. (**C**) Representative force (F) *vs.* end-to-end distance curves for WT (black) and S47P (red) obtained from CG-SMD. Three unfolding peaks are highlighted as peak1 (cyan), peak2 (green), peak3 (orange). (**D**) Distributions of the unfolding forces (*F_u_*) and the corresponding gain in contour lengths (Δ*L_c_*) are compared for WT (black) and S47P (red) in a scattered plot. (**E**) Schematic representation of the two unfolding pathways for WT as observed in CG-SMD. The high-force pathway or pathway 1 is labeled as shearing and the low-force pathway or pathway 2 is represented unzipping. The color code in the WT chain is maintained from figure 2B to highlight the strands and helices. Contact maps are shown alongside each step to highlight the evolution of intramolecular contacts.

The bWSME model predicts a slower folding rate for mutant compared with the WT. To test this experimentally, we performed *in vitro* folding kinetics as a function of the denaturant guanidinium hydrochloride (GdmCl) using a stopped-flow fluorescence setup. We obtain bi-exponential folding kinetic traces for both the WT and the mutant, with a linear dependence of the logarithm of the fast ($k_{\;ff}^{0}$) and a slow ($k_{sf}^{0}$) rates on the denaturant (Supplementary Figure S8). The fast and slow-folding rates for the WT in the absence of denaturant are 93.7 s^−1^ and 9.9 s^−1^, respectively (Figure 3B). For mutant, the fast-folding rate ($k_{\;ff}^{0}$ = 6.2 s^−1^) is 15 times slower and the slow-folding rate ($k_{sf}^{0}$ = 0.4 s^−1^) is 25 times slower than the WT (Figure 3B). It is interesting to note that the amplitudes of both fast and slow rates vary dynamically with the [GdmCl]. For the WT, the amplitude corresponding to fast-phase dominates at low denaturant concentrations and seems to cross-over with the amplitude of the slow-phase at 1.45 M of GdmCl (Figure 3A). A similar feature can be observed for S47P with the faster component is dominating within our measurement range.

However, the overall contribution of the fast component is lower than the WT and no cross-over is observed. In other words, the relative contribution of the slow-folding component is more for mutant than the WT. In conventional refolding experiments, the amplitude of the slower phase (that generally corresponds to the rate determining step) decreases with increased destabilization resulting from shifts in equilibrium towards the unfolded ensemble. We observe the exact opposite for the WT here where the slow component starts dominating at higher denaturant concentration. This dynamic switch of amplitudes and hence the corresponding rates with denaturant is suggestive of parallel folding pathways. The relatively higher amplitude of the slow component in the mutant folding indicates that it prefers the slow-folding pathway relatively more than the WT. This in turn deaccelerates the overall folding significantly (10^2^ times than WT) (Supplementary Figure S8). It is interesting to note that while the bWSME model predicts the slow folding for the mutant accurately, it does not attribute the slower folding to parallel folding pathways (Figure 1E).

### *In silico* force pulling of individual molecules identifies multi-step unfolding

To explore the presence of parallel folding pathways and the conformational changes associated with it we performed coarse-grained steered molecular dynamics (CG-SMD) simulations using a SOP model with the crystal structures of WT (4AQ8) and S47P (4AQE) (see Methods). We preferred CG-SMD over all-atom SMDs as it allowed us to reach much longer simulation times (48 ms/simulation, 9.6 s in total), perform a vastly larger number of simulations (100 each) and unfold at much slower velocities (2.5 µm/s). This is particularly important in this case as we know the unfolding follows multiple paths, requiring multiple simulations and the mutation affects long-term stability of the protein, requiring long simulations with slow pulling velocities. It is important to note that the proteins constituting the tip-link are under constant tension even in the absence of an acoustic stimulus [64]. A mutation that reduces the cross-correlated motions among constituting β-strands by the weakening of the long-range *H-*bond networks is thus expected to respond to mechanical tension differently. Exploring the differential impacts of the time-dependent forces on WT and S47P in SMD may thus be beneficial. Calculation of inter-residue distances, which is required for the structure-based approach used here, revealed subtle differences in the two crystal structures (Supplementary Figure S9) leading to a smaller number of inter-residue contacts in S47P, which could potentially alter the course of simulations. We analyzed the unfolding trajectories by plotting unfolding forces (*F_u_*) and change in contour lengths (Δ*L_c_*). In addition, we calculated contact maps for all the frames of the trajectories. In agreement with ensemble equilibrium data, the force-extension curves featured more than two-step unfolding of Cdh23 with three unfolding forces. No noticeable differences are observed between WT and mutant in the unfolding force-distributions (Supplementary material). However, a correlated plot between Δ*L_c_* and *F_u_* (Figure 3C,D, Supplementary Figure S10b) featured two distinct unfolding pathways (Figure 3D) during the second rupture event, a low-force unfolding at a peak force of 2.41 ± 0.09 kcal/molÅ and Δ*L_c_* of 10.90 ± 0.34 nm, and a high-force unfolding at a peak force of 3.75 ± 0.16 kcal/molÅ and Δ*L_c_* of 13.67 ± 0.21 nm (Figure 3C,D). This is in line with the experimental measures of the kinetic pathways. WT follows both the pathways with nearly equal probabilities (51% high-force, 49% low-force). S47P, on the other hand, predominantly follows the low-force pathway (88%).

Upon analyzing the trajectories, we observed that in Pathway 1, the high-force pathway, the unfolding starts from the N-terminus, leading to unfolding till β5 while the rest of the protein stays folded. The next step is the detachment from the C-terminus and the interactions between β5 and β8 (Figure 3E) are broken leading to the unfolding of the C-terminus part (Supplementary Movie S1, Figure 3D). Complete unfolding is achieved by the disruption of contacts between β5, β2, β6, and β7. Interestingly, this pathway is predicted by the bWSME model wherein the N-terminal half unfolds first followed by the C-terminal half of the protein. In Pathway 2, the lower force pathway, there is the simultaneous release of the N and C-termini leading to unfolding till β3 from the N-terminus and detachment of β9 from β8 on the C-terminus (Supplementary Movie S2, Figure 3E). This is followed by the disruption of β3-β7 on the N-terminus and β5-β8 on the C-terminus. The last step in Pathway 1, is the disruption of contacts between β5, H2, β6, and β7. The observation that S47P follows Pathway 2 (where the C-terminus unfolds early) is consistent with the lower number of inter-residue contacts made by the C-terminus and the results from MD simulations which showed a stronger *H*-bond network due to the interaction between S47 and E81. It is interesting to note here that while the second step involves the detachment of β8 from β5 in both the pathways, the previous detachment of β9 from β8 converts the sliding detachment to unzipping leading to unfolding at lower force (Figure 3E). Importantly, the correlated motion between β8 and β9 is lost in the mutant, a resultant of the loss of *H-*bonding network between E81 (in β8) and K94 (in β9). Overall, our SMD results further highlight the possibility of the parallel kinetic pathways for both WT and S47P.

### The specificity of S47

**Figure 4.**
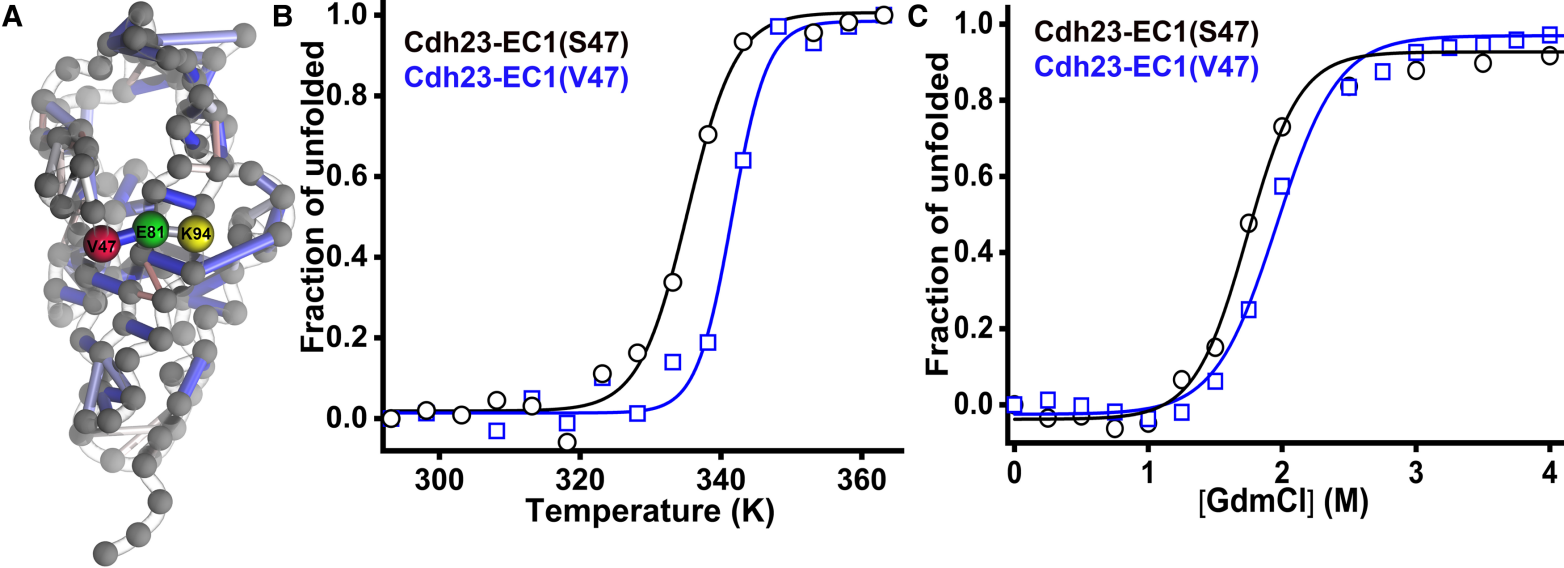
Structural and thermodynamic properties of Cdh23-EC1(V47). (**A**) Bead-and-stick representation of the intramolecular *H-*bond networks for Cdh23-EC1(V47). Colored tubes indicate the *H*-bonds between residues while the colors represent the degree of persistence (Blue (1)-White (0.62)-Red (0.25)). (**B**,**C**) Thermal (**B**) and chemical unfolding (**C**) of the wildtype variants of Cdh23 as monitored by far-UV CD and intrinsic tryptophan fluorescence, respectively. The data are normalized to estimate unfolded populations (see Methods). The solid lines are fits to a two-state model for a visual guidance.

How specific is the S47P mutation? To evaluate this, we resorted to the server Frustratometer [65] that provides a first look at the degree of frustration of specific amino acids at specific positions. We find that S47 in Cdh23 contributes to minimally frustrated interactions (Supplementary Figure S11). As per the principle of minimal frustration [65], the majority of other amino acid pairs than Ser at the 47th position of Cdh23 would introduce unfavorable interactions. True to this observation, Pro47 enhances the frustration (Supplementary Figure S11). However, Ser47, though conserved in most species, is evolutionarily replaced with Val(V) in Zebrafish, Callorhinchus milii (fish), Gekko japonicus (reptiles), anser cygnoides domesticus (Swan goose), Alligator mississippiensis (crocodile reptile), Gallus gallus (ave) (Supplementary Figure S2). Many of these species with the V47 possess better sensitivity in hearing than humans, specifically at a lower frequency. Some of these species also evolutionarily fulfill the need of hearing at high underwater pressure or low air pressure. We find that V47 in Cdh23-EC1 leads to the formation of four additional minimally frustrated contacts with residues E49, A51, V90, T92 while maintaining the two formed by S47 (Supplementary Figure S11). V47 in Cdh23 thus can facilitate the formation of favorable native interactions during folding and may steer more robust folding than the S47-variant. We thus considered Cdh23-EC1(V47) as an antipode to the PHL mutant (S47P) and investigated the effect of Val47 on Cdh23. Since the crystal structure for the V47-variant is not available, we modeled the protein from the WT structure (4AQ8) and mutated using PyMOL. We noticed that the native *H-*bond network is restored and even strengthened by valine at position 47. V47 maintains the *H*-bond with E81 as in S47 (Figure 4A) but with a higher persistence (54%), indicating further stabilization of the *H-*bond. The stabilization of V47-E81 interactions further strengthens the E81-K94 interaction in Cdh23-EC1(V47).

To measure the thermodynamic stability differences in these two evolutionarily different proteins, we performed temperature- and denaturant-induced unfolding studies using CD and tryptophan fluorescence as before. We observe a higher *T_m_* for the V47-variant, indicating a better thermal resistance towards unfolding for V47 than S47-variant (Figure 4B). Similarly, we observe a higher *C_m_* for V47 than S47 from chemical denaturation experiments (Figure 4C). Clearly, V47-variant is more compact with denser inter-residue interactions and thermodynamically more stable than S47-variant. Thus, the substitution to Val may not be random, but could rather arise from an evolutionary demand for sustaining in high underwater hydrostatic pressure [66].

## Discussion

In progressive hearing loss (PHL), the onset of hearing loss starts much early and drives towards complete loss at a relatively faster rate than normal [16]. Our study focuses on a mutation that maintains the native structure and function of the WT from birth but, however, deteriorates with aging at an aggressive rate and causes PHL. It is important to note that the mutation of the protein is in the extracellular region, and the site is not associated with cellular signaling. Loss of function with aging in PHL is thus arising from the gradual changes in the physical properties of the protein. We connect the differences in the biophysical properties of the mutant with the disease and subsequently propose a predictive model for ARHL with the WT protein. In this work, we evaluated the properties of the WT and mutant protein over a large parametric space spanning the equilibrium and dynamic properties. We report a lower thermodynamic stability, remarkable loss of cross-correlated motions in β-strands, and a significantly slower rate of folding with mutation. While the lower thermodynamic stability makes the mutant more vulnerable to the heat-shock, the lack of correlated motions in the mutant reduces its tenability. Less correlated motions in β-strands makes the mutant sensitive to perturbations, thus incurring gradual deformations with sound or other external stimuli. The slow refolding is an experimental manifestation of the reduced cross-correlated motions. Our SMD simulations also indicate a low-force unfolding pathway as most preferred for the mutant, indicating that the mutant is more prone to external perturbations, either isotropically arising from thermal energy change or anisotropically arising from loud noise.

Long-range electrostatic interactions, predominantly *H*-bond interactions sculpt the cross-correlated motions among β-strands in a protein [48]. The extent of electrostatic interactions is delicately dependent on the ionic strength of the media. It is, therefore, expected that changes in the inner ear fluid composition with age, will alter the cross-correlated motions among β-strands in Cdh23 and carve gradual deformations in the protein, leading to hearing loss. The fluctuations in the inner ear fluid composition with aging are common, e.g. depletion in calcium concentrations due to aberrant calcium homeostasis [67], alterations in the glucose concentration due to reduced metabolic rates [68]. We, therefore, propose that the gradual change in the inner ear fluid composition with aging hinders the cross-correlated motions among β-strands in the Cdh23 by altering the inter-residue interactions, and makes the protein more susceptible to deformations under sound stimuli.

Tip-link proteins regularly experience mechanical tension from sound stimuli. As shock resistors, the correlated motions in the β-rich domains help tip-links to accommodate the conformational changes and absorb or dissipate tension. Loss in correlated motions with time from the break in *H-bond* network and loss of intra-residue interactions gradually abolish the shockproof ability, leading to hearing loss with aging. Our work highlights such loss in correlated motions can modulate both stability (weakening native interactions) and rates (slower rates) contributing to progressive hearing loss. What triggers such loss in the interaction network under physiological conditions with time is not clear from here. One likely needs to consider the external environmental noise and internal noise such as the effect of drugs, metabolites, minerals etc. to identify the perturbations and their consequences. Our work thus lays the foundations for understanding hearing loss from the perspective of basic biophysical factors.

## Data Availability

All data generated or analyzed during this study are included in this published article (and its supplementary information files).

## Author notes

These authors contributed equally: S.G., A.S. and G.S.S.

## Abbreviations

ARHL: age-related hearing loss
CD: circular dichroism
CG-SMD: coarse-grained steered molecular dynamics
DSC: differential scanning calorimetry
MD: molecular dynamics
PHL: progressive hearing loss
SOP: self organizing polymer
WT: wildtype
