# Supplementary material for: Weakening of interaction networks with aging in tip-link protein induces hearing loss
Source: Biochem J. 2021 Jan 13;478(1):121–34. doi: 10.1042/BCJ20200799 (PMC7813477; doi:10.1042/BCJ20200799)
Supplement: Supplementary Figures S1-S11 [file BCJ-478-121-s1.pdf]

Supporting Information

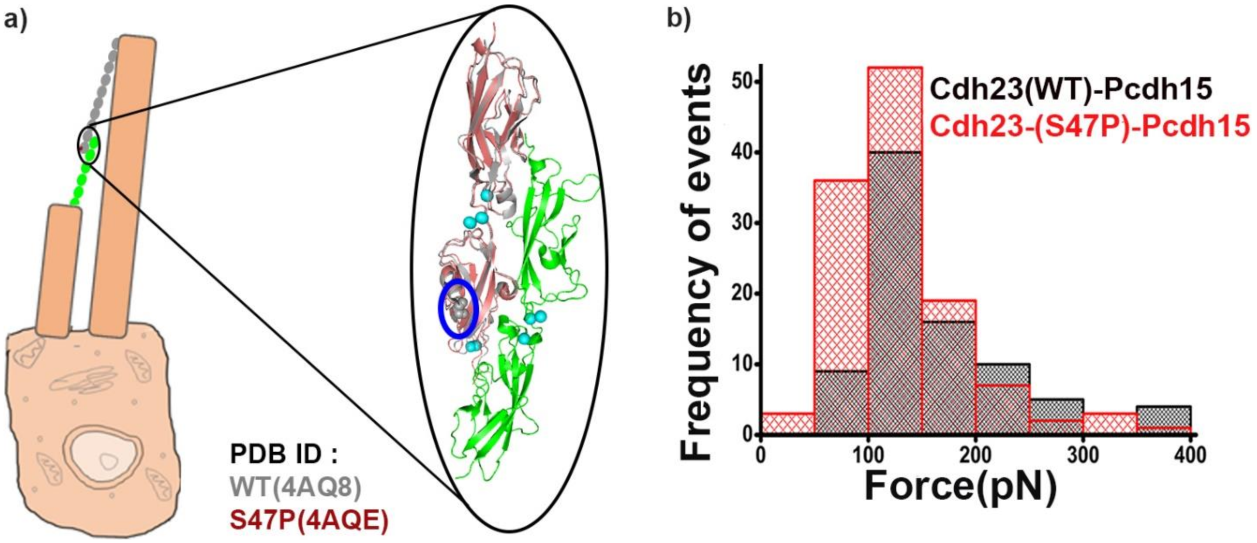

SI Figure 1. Tip-link complex. a) Cartoon representation of tip link complex interacting through two outermost extracellulr domains of Cdh23(gray) and Pcdh15(green) zoomed in black circle (left). The secondary structure of the tip link complex as solved by X-ray crystallography is shown in zoomed view. The 47th residue of Cdh23 is marked in blue ellipse. **b)** The unbinding force-distributions for the WT (black) and mutant (red) complexes are shown as recorded using SMFS with AFM at a pulling velocity of 750 nm/s. We observed no noticeable differences in the

distributions of unbinding forces between WT and mutant complexes, recorded at three different pulling velocities.

```
Hs__QVNRLPFFTNHFFDTYLLISEDTPVGSSVTQLLAQDMDNDPLVFGVSGEEASRFFAVEPDTGVVWLRQPLDRETKSEFTVEFSVSDHQGVITRKVNIQVGD
Fc__QVNRLPFFTNHFFDTYLLISEDTPVGSSVTQLLARDMDNDPLVFGVSGEEASRFFAVEPDTGVVWLRQPLDRETKSEFTVEFSVSDHQGVITRKVNIQVGD
Clf_QVNRLPFFTNHFFDTYLLISEDTPVGSSVTQLLARDMDNDPLVFGVSGEEASRFFAVEPDTGVVWLRQPLDRETKSEFTVEFSVSDHQGVITRKVNIQVGD
Ea__QVNRLPFFTNHFFDTYLLISEDTPVGSSVTQLLARDMDNDPLVFGVSGEEASRFFAVEPDTGVVWLRQPLDRETKSEFTVEFSVSDHQGVITRKVNIQVGD
Bt__QVNRLPFFTNHFFDTYLLISEDTPVGSSVTQLLARDMDNDPLVFGVSGEEASRFFAVEPDTGVVWLRQPLDRETKSEFTVEFSVSDHQGVITRKVNIQVGD
Mm__QVNRLPFFTNHFFDTYLLISEDTPVGSSVTQLLARDMDNDPLVFGVSGEEASRFFAVEPDTGVVWLRQPLDRETKSEFTVEFSVSDHQGVITRKVNIQVGD
Rn__QVNRLPFFTNHFFDTYLLISEDTPVGSSVTQLLARDMDNDPLVFGVSGEEASRFFAVEPDTGVVWLRQPLDRETKSEFTVEFSVSDHQGVITRKVNIQVGD
Ss__QVNRLPFFTNHFFDTYLLISEDTPVGSSVTQLLARDMDNDPLVFGVSGEEASRFFAVEPDTGVVWLRQPLDRETKSEFTVEFSVSDHQGVITRKVNIQVGD
Pt__QVNRLPFFTNHFFDTYLLISEDTPVGSSVTQLLARDMDNDPLVFGVSGEEASRFFAVEPDTGVVWLRQPLDRETKSEFTVEFSVSDHQGVITRKVNIQVGD
Ef__QVNRLPFFINHFFETYLLISEDTPVGSSVTQLLARDMDNDPLVFGVSGEEASRFFAVEPDTGVVWLRQPLDRETKSEFTVEFSVSDHQGVITRKVNIQVGD
Lv__QVNRLPFFINHFFDTYLLISEDTPVGSSVTQLLARDLDNDPLVFGVSGEEASRFFAVEPDTGVVWLRQPLDRETKSEFTVEFSVSDHQGVITRKVNIQVGD
Acd_WGNRLPYFINYFFDTYLLINEDTPVGSSVTQLLARDLDNDPLVFGVVGEEASRFFAVESVTGVVWLRQPLDRETKSEFTVEFSVSDSQGVIKGTVNIQVGD
Gg__QGNRLPYFINYFFDTYLLINEDTPVGSSVTQLLARDLDNDPLVFGVVGEEASRFFAVESVTGVVWLRQPLDRETKSEFTVEFSVSDSQGVIKGTVNIQVGD
Am__LSNRLPYFINYFFDTYLLINEDTPVGSSVTQLIARDLDNDPLVYGVVGEEASRFFAVESVTGVVWLRQPLDRETKSEFTVEFSVSDSQGVIKGTVNIQVGD
Cm__LSNRLPYFINYFFDTYLLINEDTPVGSSVTQLLARDLDNDHLVFGVVGEEASRFFAVESVTGVVWLRQPLDRETKSEFTVEFSVSDSQGVIKGTVNIQVGD
Gj__LSNRLPYFINYFFDTYLLINEDTPVGSSVTQLLARDLDNDPLVFGVVGEEASRFFAVESMTGVVWLRQPLDRETKSEFTVEFSVSDSQGVIKGSVNIQVGD
Dr__-MNQPPRFQNYFFQSYLLVYEDTPVGTSITQLQAVDPDGEPLIFGVVGEEAMRYFAVQGTTGVVWLRQPLDREAKSEMQVEFTVSDSQGVVKDTVNIQIGD
```

Homo sapiens (Hs),Felis catus (Fc),Canis lupus familiaris (Clf),Equus asinus (Ea),Bos taurus (Bt),
Mus musculus (Mm),Rattus norvegicus (Rn),Sus scrofa (Ss),Pan troglodytes (Pt),Eptesicus fuscus (Ef),
Lipotes vexillifer (Lv),Anser cygnoides domesticus (Acd),Gallus gallus (Gg),
Alligator mississippiensis (Am),Callorhinchus milii (Cm),Gekko japonicus (Gj),Danio rerio (Dr)

SI Figure 2. **Sequence alignment of Cdh23-EC1 for different species.** Position 47 is highlighted that is populated by Serine in some species and Valine in others.

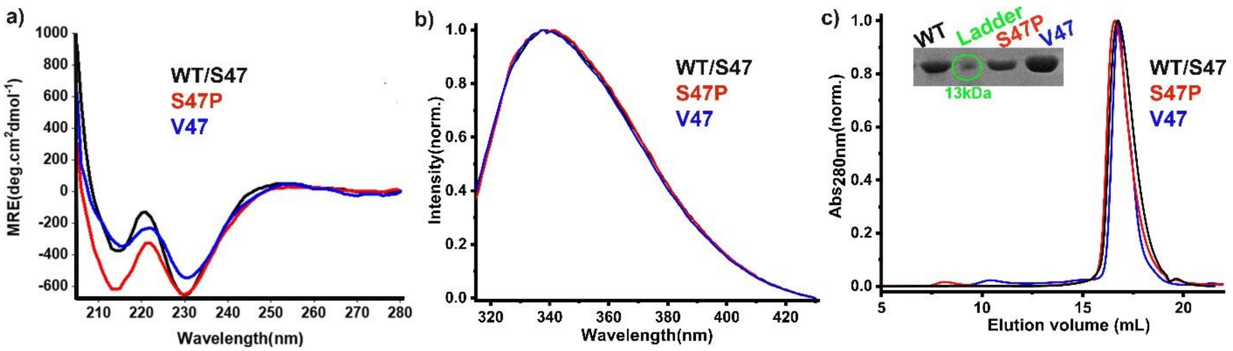

SI Figure 3. Spectroscopic comparisons of Cdh23 variants. a) FUV CD, b) Fluorescence emission spectra, c) Chromatogram from SEC are shown here. Inset in Figure (c) shows the SDS PAGE of WT/S47 (black), S47P (red), and V47 (blue) where the ladder at 13 kDa is encircled in green.

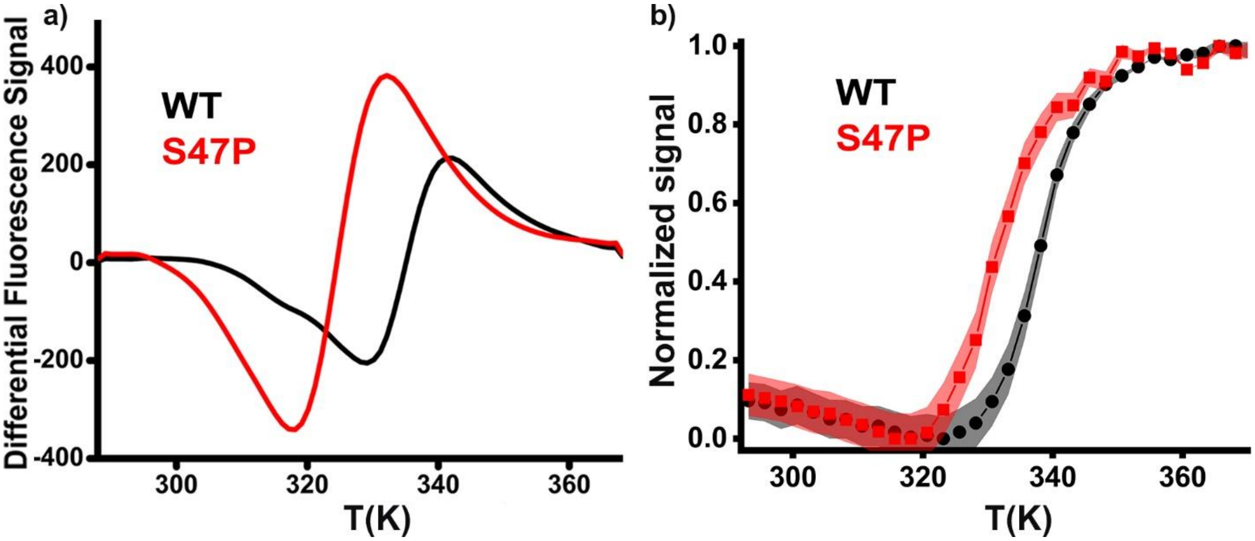

SI Figure 4. **Thermal unfolding of WT and S47P. a)** A comparative plot monitoring the exposure of hydrophobic surface upon thermal unfolding using Differential Scanning Fluorimetry (DSF) for WT (black) and S47P (red). Sypro Orange dye is used as a fluorophore that exhibits an emission maximum at 570 nm on binding to the hydrophobic patches as the protein unfolds. We observed a lower melting temperature (T_m) for the S47P mutant, i.e., 316 K for the mutant versus 326 K for the WT. **b)** Thermal unfolding of WT (black) and S47P (red) monitored from intrinsic tryptophan fluorescence are shown. Intrinsic Trp (W66) emissions also display a lower thermal denaturation(T_m) for the mutant compared to the WT. The shaded portions (black (WT) and red (S47P)) mark the standard error of mean (SEM) from experimental repeats.

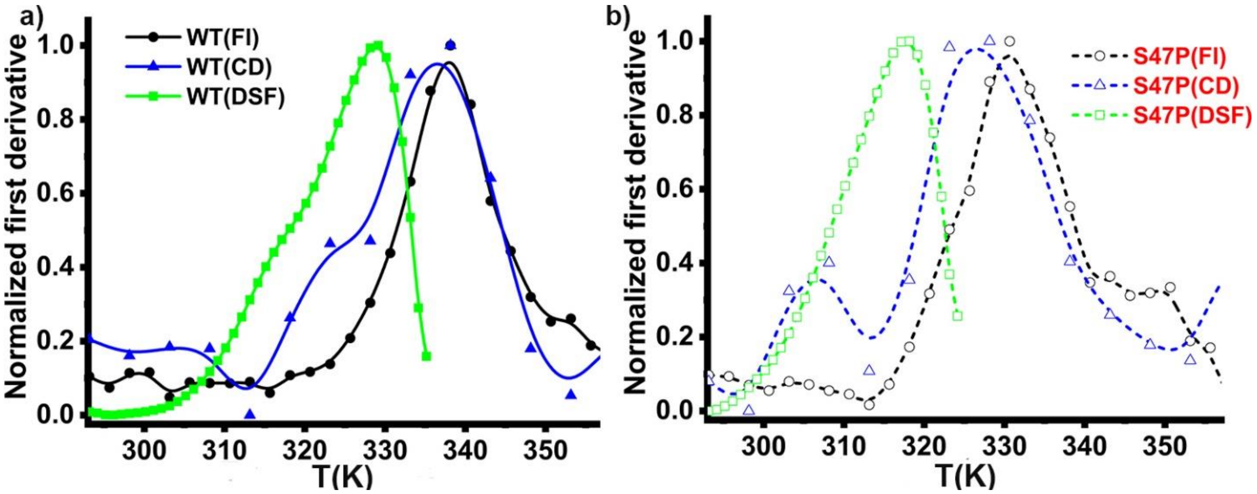

SI Figure 5. Derivatives of thermal unfolding curves for WT and S47P. a & b) Derivatives of thermal unfolding curves as monitored by Tryptophan fluorescence (Fl) (black), far-UV CD (blue) and DSF (green) are shown for (**a)** WT (**b)** S47P. Differences in melting temperatures indicate that the unfolding is not a two-state process for both the cadherin variants. The solid (WT) and dotted (S47P) lines are only for visual guidance.

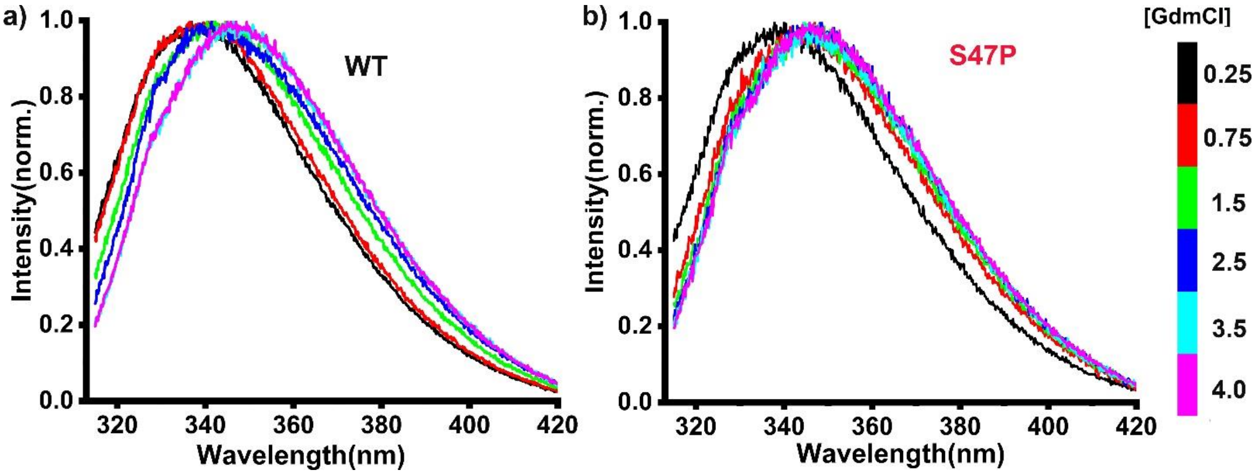

SI Figure 6. Fluorescence spectra of WT and S47P with denaturant. a & b) Normalized emission spectra of **(a)** WT and **(b)** S47P at varying [GdmCl] are shown to signal the absence of isoemissive point. The color map represents the GdmCl concentrations.

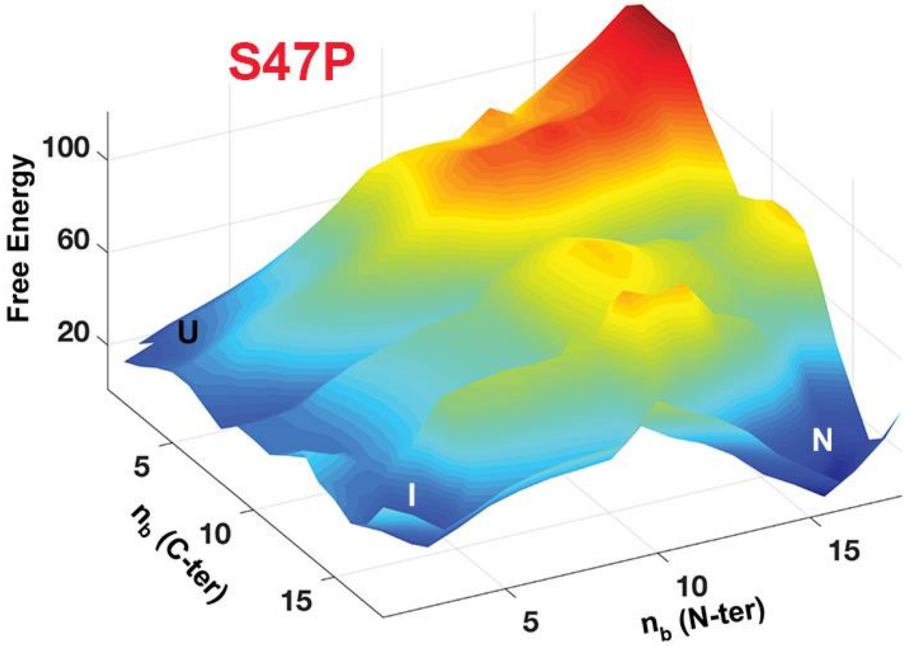

SI Figure 7. **Free energy landscape of S47P.** Free energy landscape of the S47P from the bWSME model as a function of the number of structured blocks in the N- or C-terminus. The unfolded state (U), intermediate state (I), and native state (N) is marked on the landscape.

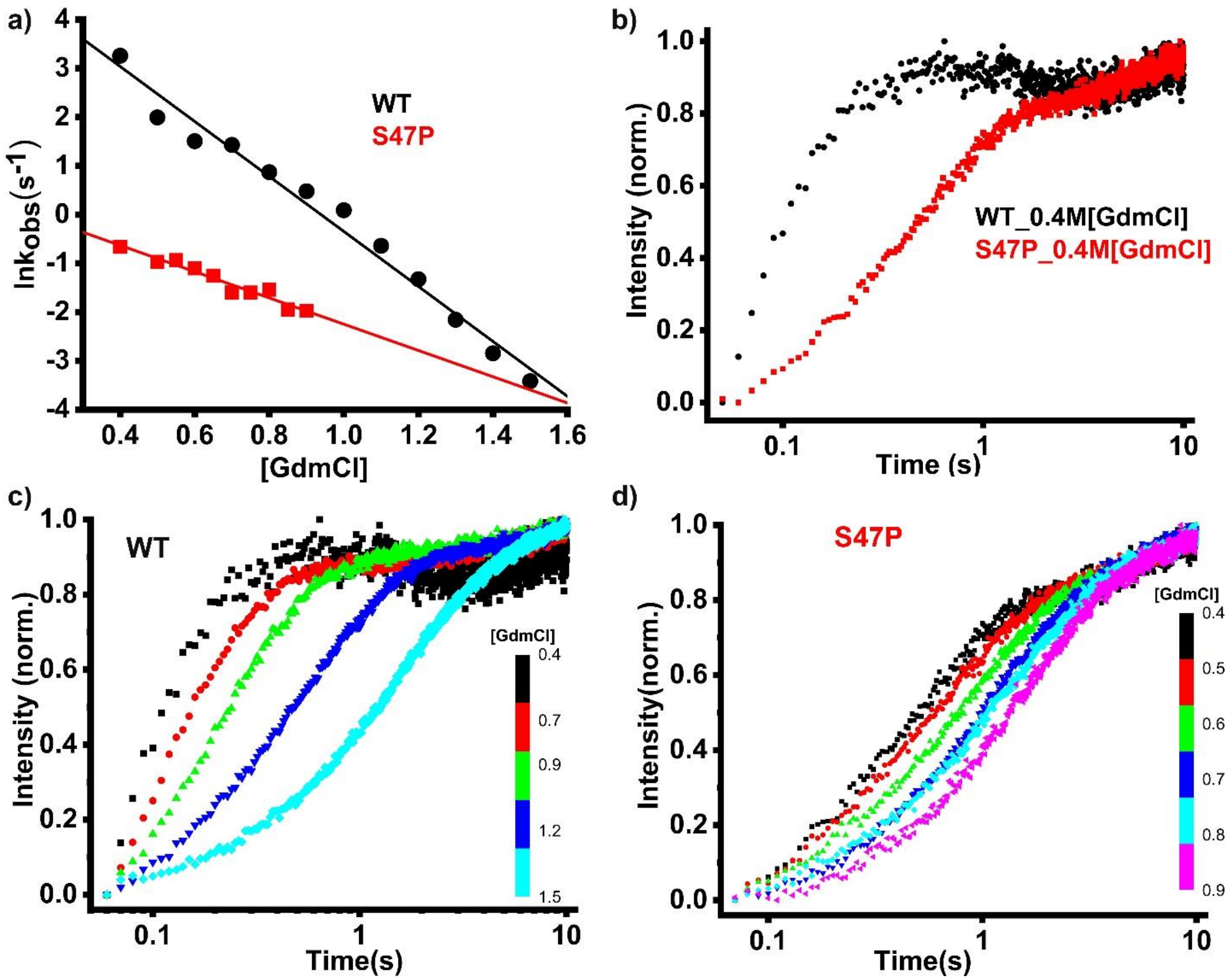

SI Figure 8. Folding rate and folding traces of WT and S47P. a) Comparision of the folding rate for WT (black) and S47P (red) at varying [GdmCl]. **b)** Comparision of the folding traces for WT (black) and S47P (red) at 0.4 M of GdmCl. **c & d)** Folding kinetics of WT (b) and S47P (c) at varying [GdmCl]. The color maps represent the GdmCl concentrations.

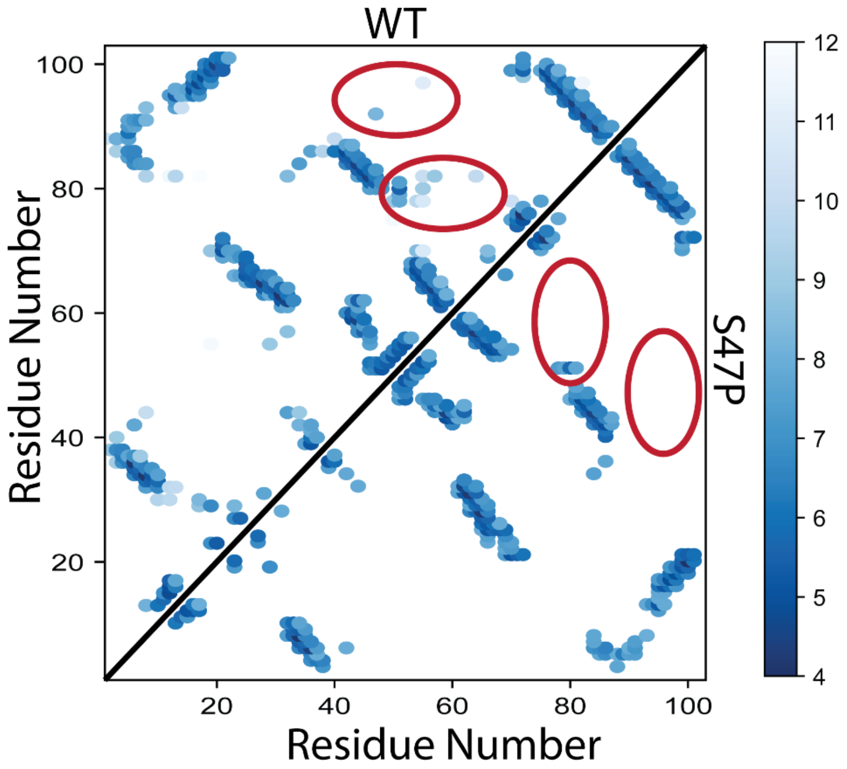

SI Figure 9. Inter-residue distances for WT and S47P. Pairwise inter-residue distances (Cα-Cα) for all residues separated by two covalent bonds for WT (upper diagonal) and S47P (lower diagonal) plotted from the topology file used for SOP-GPU simulations. The color represents the distance between the residues. The contacts present in WT which are lost in S47P are highlighted with red ovals.

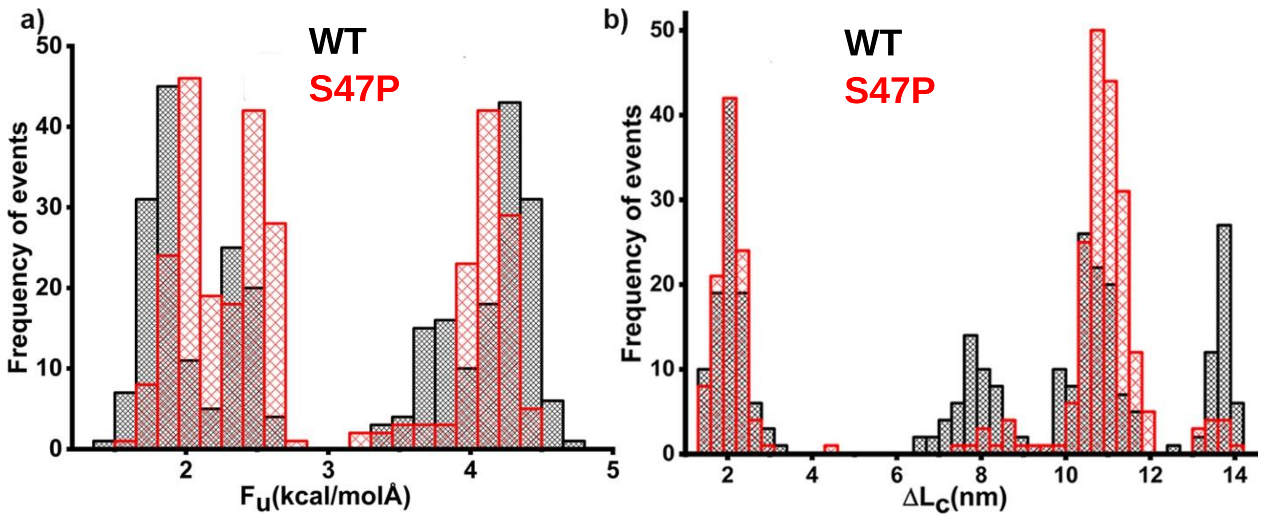

SI Figure 10. Force-unfolding of WT and S47P by CG-SMD. a) Unfolding force distributions (F_u) of WT (black) and S47P (red) are compared as obtained from CG-SMD. The WT has four distinct distributions while S47P has only three distributions. **b)** Contour length (ΔL_c) distributions of of WT (black) and S47P (red) are compared. Similar to force-distributions, WT unfolding follows four distinct distributions of contour-length whereas S47P shows only two.

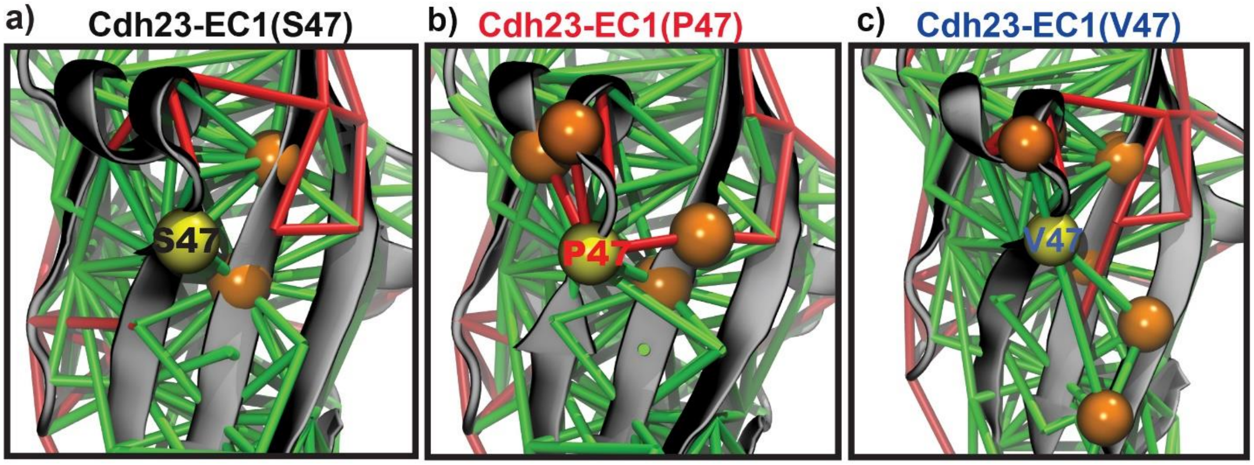

SI Figure 11. Frustration analysis for S47, P47, and V47 of Cdh23. Frustration analysis for **a)** S47, **b)** P47, and **c)** V47. The residue at position 47 is shown as a yellow sphere and all residues with which it forms a minimally or highly frustrated contacts are shown as orange spheres. The green and red cylinders represent minimally and highly frustrated contacts, respectively.

SI Movie 1 : High force pathway unfolding. Unfolding starts from the N-terminus, unfolds till β5 while the rest of the protein stays folded. The next step is the detachment from the C-terminus and the interactions between β5 and β8 are broken leading to the unfolding of the C-terminus part. Complete unfolding is achieved by the disruption of contacts between β5, β2, β6, and β7.

SI Movie 2 : Low force pathway unfolding. There is the simultaneous release of the N and C-termini leading to unfolding till β3 from the N-terminus and detachment of β9 from β8 on the C-terminus. This is followed by the disruption of β3-β7 on the N-terminus and β5-β8 on the C-terminus.
